# Glucocorticoid-Induced TNFR-Related (GITR) Protein and Its Ligand in Antitumor Immunity: Functional Role and Therapeutic Modulation

**DOI:** 10.1155/2010/239083

**Published:** 2010-09-26

**Authors:** Theresa Placke, Hans-Georg Kopp, Helmut Rainer Salih

**Affiliations:** Department of Hematology/Oncology, Eberhard Karls University, Otfried-Mueller-Str. 10, 72076 Tuebingen, Germany

## Abstract

The ability of the tumor necrosis factor receptor (TNFR) family member GITR to modulate immune responses has been the subject of multiple studies. Initially thought to be critically involved in governing functions of regulatory T cells, GITR and its ligand GITRL have meanwhile been found to modulate the reactivity of various different cell types and to influence a broad variety of immunological conditions including the immune response against tumors. Not only GITR, but also GITRL is capable of transducing signals, and the consequences of GITR-GITRL interaction may vary among different effector cell types, differ upon signal transduction via the receptor, the ligand, or both, depend on the level of an ongoing immune response, and even differ among mice and men. In this paper, we address available data on GITR and its ligand in immune responses and discuss the role and potential therapeutic modulation of this molecule system in antitumor immunity.

## 1. Introduction

Many members of the TNFR family and their ligands play an important role in proliferation, differentiation, activation, and cell death of both tumor and immune effector cells. In humans, the TNFR family member GITR was first identified in 1999 by two independent groups as orthologue of murine GITR, which had been described two years earlier as a dexamethasone-inducible molecule in T cells [1–3]. GITR is also known as AITR (Activation-Inducible TNFR family member) or TNFRSF18 and is a type I transmembrane protein with a cysteine-rich extracellular domain, the latter representing a common feature of the TNFR family. Its cytoplasmic domain exhibits close homology with that of the TNFR family members 4-1BB/CD137 and CD27 [3]. While different splice variants of GITR have been described in both men and mice ([4] and GenBank numbers NM_148901.1, NM_148902.1, NM_004195.2), detailed data on the expression profile of the various splicing variants are not available as of yet.

Human GITR ligand (GITRL, TNFSF18, AITR ligand) was identified simultaneously with its receptor [1, 2], whereas its murine orthologue was cloned in 2003 [5, 6]. Like most TNF family ligands, it is a type II transmembrane protein. Available data suggest that human GITRL is a trimer, but can also be a monomer or assemble in other multimeric structures, whereas murine GITRL associates as a dimer [7–10]. In humans, also a soluble form of GITRL (sGITRL) has been detected on the protein level [11–13]. The mechanism by which the soluble form of GITRL is produced, that is, by shedding of the surface-expressed form, for example, due to the activity of metalloproteases or upon alternative splicing, is still unclear.

## 2. GITR and GITRL Expression Pattern

On human and murine CD4+CD25+ regulatory T cells (Treg), high levels of GITR can be detected in steady-state with a further increasing expression upon stimulation [14–18]. Effector CD4+ and CD8+ T cells express GITR constitutively at low levels, but rapidly upregulate GITR expression upon activation [1–3, 15, 17, 19–25]. In mice, expression of GITR has also been detected in B cells, natural killer (NK) cells, NKT cells, granulocytes, and macrophages [5, 15, 25–28], whereas in humans GITR expression has been described in macrophages and NK cells [27, 29–33]. On the latter, GITR expression is, alike in T cells, upregulated following activation. Some nonhematological tissues like skin and lung have also been found to express GITR mRNA in mice and humans [1, 34]. 

Of note, some ex vivo studies revealed differential GITR expression patterns on T cells dependent on disease state. Li et al. reported that CD4+ T cells of patients with noninfectious uveitis express higher levels of GITR than those of healthy controls, and expression of GITR correlated with disease course [18]. In HIV-infected humans, higher baseline expression of GITR on CD4+ T cells compared to healthy donors was observed [35]. In patients with Wegener's granulomatosis, GITR expression on CD4+CD25+ T cells correlated with disease activity [36]. Lee et al. reported elevated expression of GITR in patients with active systemic lupus erythematosus as compared to patients with inactive disease [37], and children with type I diabetes displayed diminished mRNA levels of GITR in Treg as compared to controls [38]. These data indicate that GITR expression and likely also function may depend on the activity levels of the respective immune effector cell populations.

The cognate ligand of GITR has, in men, been found in endothelial cells, dendritic cells (DC), macrophages, and cells of the eye and can be upregulated on the latter by proinflammatory cytokines [1, 2, 39]. Human monocytes were found to transiently up-regulate GITRL upon stimulation [40]. Murine GITRL has been detected on DC, monocytes, macrophages, B cells, endothelial cells, osteoclasts, and microglia cells [5, 6, 16, 23, 41, 42]. GITRL is absent from resting T cells, but the data whether it is expressed on T cells following activation are at least partially conflicting [6, 16, 22]. We demonstrated recently that various tumor cell lines as well as primary solid tumors of different histological origin and patient leukemia cells express substantial levels of GITRL, and elevated levels of sGITRL are present in sera of patients with various malignancies [11, 31, 43]. Moreover, we found that GITRL is upregulated on megakaryocytes during maturation resulting in substantial GITRL expression by platelets ([44] and unpublished data).

## 3. Consequences of GITR Activation in T Cells

As of now, most functional studies with GITR focused on T cells. The initially described function of GITR was its ability to protect T cells from activation-induced cell death (AICD) [1, 3]. Subsequently, several groups demonstrated that GITR triggering by agonistic antibody, by GITRL expressed on transfectants, or upon addition of GITRL in soluble form abrogates suppression of murine CD4+CD25+ Treg [5, 6, 14–16, 45, 46]. Of note, human Treg were found to maintain their suppressive function after GITR stimulation with antibody, by GITRL transfected into DC or recombinant GITRL [47, 48]. While this indicates that GITR may mediate differential effects in mice and men, it needs to be considered that in the mouse studies freshly isolated Treg were used, while polyclonal populations of CD4+CD25+ T cells or isolated Treg from cancer patients were employed in the human system. Thus, differences in the settings used to stimulate GITR and/or the investigated cell populations may have influenced the functional consequences of GITR-GITRL interaction. Besides modulating Treg reactivity, the ability of GITR to mediate costimulatory signals in responder T cells receives increasing attention, and GITR triggering by sGITRL, agonistic antibody, or GITRL expressed on transfectants has been shown, among others, to increase T cell proliferation and cytokine production both in the human and murine systems [6, 13, 15, 16, 19, 24, 45, 46, 48–52]. Of note, the effects of GITR costimulation seem to depend on the strength of the first signal, indicating that the consequences of GITR triggering may be influenced by the level of immune activation [19, 50].

Analyses of GITR functions in mouse models were employed to study the role of this molecule both with regard to its physiological function and its influence in various diseases. GITR^−/−^ mice are available in several mouse strain backgrounds; they are viable with an apparently healthy phenotype and without significant deficiencies in immune cell development or numbers [53, 54]. Their T cells show a phenotype characterized by increased proliferation and a higher sensitivity to AICD, and their Treg display suppressive activity comparable to that of wild type mice. The percentage of CD4+CD25+ cells in GITR^−/−^ mice is slightly decreased [16, 54, 55]. In line, spleens of transgenic C57BL/6 mice expressing GITRL cDNA under the control of the CD19 promotor contained up to three times more CD4+foxp3+ T cells as compared to wild type mice, suggesting a role of GITR in the amplification of Treg [56].

Despite the initial view of GITR as a direct inhibitor of Treg function, data obtained in GITR^−/−^ mice indicate that induction of responder T cell resistance to Treg-mediated suppression may be a central mechanism underlying the effects observed upon GITR signaling [16]. Various experimentally induced autoimmune diseases take an attenuated course in GITR^−/−^ mice. With regard to collagen-induced arthritis, Sv129 GITR^−/−^ mice displayed less joint inflammation and bone erosion than wild type controls. Additionally, the authors reported lower concentrations of inflammatory mediators and suggested that GITR^−/−^ mice show decreased inflammatory responses due to reduced costimulation of effector T cells and sustained suppressive capacity of Treg [55]. Candida albicans infection was found to take an attenuated course in the same GITR^−/−^ mice, and similar findings were obtained in models of acute lung inflammation upon pleural injection of carrageenan and lung injury following intratracheal installation of bleomycin [53, 57, 58]. Moreover, treatment with agonistic GITR antibody induced or exacerbated autoimmune gastritis, collagen-induced arthritis, and experimental autoimmune encephalomyelitis (EAE) in wild type mice and autoimmune diabetes in nonobese diabetic (NOD) mice due to stimulation of responder T cells [15, 24, 59, 60]. Of note, the B cell-specific GITRL transgenic mice described above showed delayed onset of EAE [56] indicating that experimental procedures, that is, GITR triggering by antibody or enforced expression of GITRL may be relevant for disease modulation. Agonistic GITR antibody also enhanced CD4+ and CD8+ T cell immune responses against herpes simplex virus type 1 as exemplified by increased numbers and cytotoxicity of virus-specific cytotoxic lymphocytes in BALB/c and C57BL/6 mice [23, 61]. In addition, treatment with agonistic GITR antibody enhanced effector T cell responses and reduced parasite burden in C57BL/6 mice infected with Leishmania donovani [62]. Heart-transplanted CBA mice show increased graft rejection, progressive decline in contractile function, and increased coronary artery vasculopathy if treated with anti-GITR, which was, in this study, not due to stimulation of CD4+ or CD8+ effector T cells [63]. Two groups reported on the effect of agonistic anti-GITR antibody in models of graft versus host disease (GvHD). Kim and coworkers reported that GITR stimulation converted chronic to active GvHD, ameliorated disease symptoms, and affected survival in (C57BL/6xDBA/2)F_1_ mice with chronic GvHD upon transfer of DBA/2 parental cells, and this was due to modulation of effector T cells [20, 64]. Muriglan et al. found that GITR stimulation enhanced alloreactive CD8+ T cell proliferation and function, but decreased reactivity of alloreactive CD4+ T cells. BALB/c mice transplanted with T cell-depleted C57BL/6 bone marrow allografts receiving CD8+ donor T cells displayed increased GvHD morbidity when treated with agonistic antibody, while recipients of CD4+ T cells showed a significant decrease in GvHD upon GITR triggering [65]. Taken together, the available data indicate that GITR may not only mediate different effects in Treg and responder T cells, but also differentially affects functions of CD4+ and CD8+ effector T cells, and the consequences of GITR signaling seem to be largely dependent on the level of the ongoing immune response. It should be noted that, in the majority of models studying the effects of GITR stimulation with agonistic GITR antibody, the observed effects were attributed to activation of effector T cells and not to abrogation of the suppressor function of Treg. However, the discussion whether immune activation and reversal of T cell suppression by GITR activation is due to effects on Treg, responder T cells, or both is still ongoing (for an excellent review see [66]).

## 4. Consequences of GITR Activation in Non-T Cells

In line with its expression pattern, GITR also influences the reactivity of various other cell types than T cells. Macrophages from both mice and humans were found to up-regulate ICAM-1 following stimulation with anti-GITR antibody subsequently resulting in aggregation and adhesion, and produced enhanced levels of MMP-9, TNF, IL-8, and MCP-1. Notably, recombinant GITRL induced weaker cytokine responses in cell lines than the antibody or even failed to stimulate cytokine production and ICAM-1 regulation in primary macrophages [27, 29]. 

Hanabuchi and coworkers reported activation of human NK cells after coculture with plasmacytoid DC (pDC), which was decreased by addition of an anti-GITRL antibody. They also reported that cytotoxicity and IFN-*γ* production of NK cells were increased in cultures with GITRL-expressing transfectants [32]. In contrast, we found that GITR triggering by GITRL expressed on or released as soluble form by transfectants reduced NK cell cytotoxicity and cytokine production. NK reactivity was restored by blocking GITR with anti-GITR antibody or neutralization of GITRL by a GITR-Ig fusion protein [11, 31, 43]. Our results that GITR inhibits the reactivity of human NK cells were subsequently confirmed by a third group reporting that GITR triggering reduced NK reactivity and proliferation. Of note, these investigators did, in contrast to our findings, observe increased NK cell apoptosis upon GITR triggering [33]. The discrepancy among the findings of the three groups studying GITR function in human NK cells is most likely due to differing experimental settings (e.g., culture conditions, source of NK cells, techniques to study NK reactivity, etc.) and/or reagents. In our hands, the anti-GITR antibody, but not the anti-GITRL antibody, used by Hanabuchi and coworkers was capable to block binding of GITR-Ig to GITRL-expressing transfectants and thus receptor-ligand interaction. Moreover, cytotoxicity and IFN-*γ* production of NK cells were enhanced by the presence of the anti-GITR antibody in cultures with the GITRL-transfectants, but not in cultures with the mock controls, which further confirmed its blocking capacity. The antibody did not directly alter NK reactivity, as it had no effect in cultures of NK cells with GITRL-negative targets or on cytokine-induced NK effector functions. In addition, no effect of the antibody was observed in experiments with GITRL-positive target cells using GITR-negative NK92 NK cells as effectors. Together, these data excluded that the anti-GITR antibody employed in our experiments had agonistic properties and confirmed that its effects in functional experiments were in fact due to blocking GITR-GITRL interaction. It seems possible that coculture of GITRL-expressing cells like pDC with GITR-expressing NK cells or with potentially agonistic GITRL antibody for longer time periods, like in the work of Hanabuchi et al., might induce reverse signaling into the GITRL-expressing cells which, in turn, could cause altered NK cell reactivity. We further analyzed the effects of triggering of GITR on NK cells using immobilized recombinant GITRL-Ig in cultures with GITR-GITRL double-negative K562 cells. This setting, where effects of reverse signalling into GITRL-expressing targets are excluded, further confirmed the inhibitory effect of GITR on human NK cells. Of note, both immobilized GITRL-Ig and target cell-expressed GITRL were found to reduce NK cell NF-*κ*B activity. This could be prevented by the anti-GITR antibody and confirmed further that GITR mediates inhibitory signals in human NK cells.

## 5. “Reverse Signaling” via GITRL

**Figure 1:**
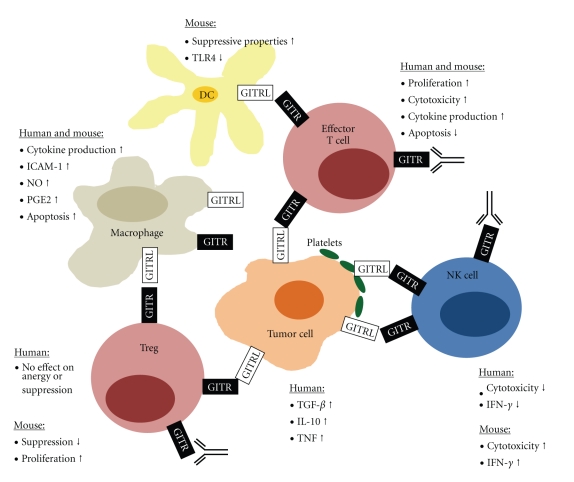
Consequences of signaling via GITR and GITRL in different cell types. Shown are effects on cellular reactions of the indicated immune effector cells and tumor cells as emerged from the multitude of available studies. It should be noted that for some of the depicted effector functions different or even opposite results have been reported in some studies.

Many membrane-bound ligands of the TNF family are able to communicate bidirectional signals. This was first postulated by Smith et al. in 1994, who suggested a physiological relevance of the short cytoplasmic domains because of their high interspecies conservation in different TNF family members [67]. Meanwhile there is an increasing body of data regarding the consequences of signaling via ligands of the TNF family, and also on reverse signaling via GITRL in different cell types. In murine DC, treatment with GITR-Ig conferred suppressive properties, and this was dependent on the presence of IFN-*α*. The authors nicely demonstrated, among others, that GITRL signaling activates indoleamine-2,3-dioxygenase in the DC and suggest that modulation of tryptophan catabolism upon GITR-GITRL interaction may represent an important mechanism of action of anti-inflammatory corticosteroids [68]. DC from GITR^−/−^ mice have been shown to display lower TLR2 and higher TLR4 expression than DC of wild type mice, and TLR4 expression was down-regulated by recombinant GITR protein confirming that GITRL signaling in fact influences DC [69]. With macrophages, multiple studies revealed that signals via GITRL modulate their activity in both mice and humans. Enhanced release of inflammatory mediators such as, MMP-9, TNF, IL-1*β*, IL-8, and MCP-1, and increased expression of ICAM-1 and cellular aggregation have been reported upon GITRL signaling [30, 70]. In addition, inducible nitric oxide (NO) synthetase and cyclooxygenase-2 are induced in murine macrophages upon stimulation with GITR, which results in enhanced secretion of NO and prostaglandin E_2_ [71, 72]. On the other hand, GITR was found to cause apoptosis and G1 phase arrest in murine macrophages upon binding to GITRL [73]. Of note, not only healthy but also malignant cells of various origins release immunomodulatory cytokines such as, TGF-*β*, IL-10, and TNF upon GITRL signaling [31, 43]. Together, these findings on an immunomodulatory function of GITRL signaling indicate that GITR-GITRL interaction may cause multiple different effects depending on the involved cell types (see also Figure 1), which may serve to explain seemingly contradicting results of different studies.

## 6. Role of the GITR-GITRL Molecule System and Its Modulation in Antitumor Immunity

In recent years, the role of GITR and its ligand in tumor immunology and especially the possibility to therapeutically modulate this molecule system as a means for treating cancer has received considerable interest. Calmels et al. injected immunocompetent C57BL/6 mice with B16-F0 tumor cells. When tumors were palpable, the authors injected adenovirus vectors leading to overexpression of membrane-bound and soluble GITRL by the tumors. This resulted in enhanced tumor infiltration by CD4+ and CD8+ T cells. Moreover, expression of membrane-bound and soluble GITRL led to a clear reduction of tumor volume and increased animal survival without inducing autoimmunity, and the investigators further reported that tumor-expressed GITRL enhanced proliferation of CD4+ and CD8+ effector T cells in the presence of anti-CD3 in vitro [49]. Similar results were obtained by other investigators. Cho and coworkers inoculated GITRL-transfected poorly immunogenic CT26 cells into BALB/c mice and reported that GITRL expression delayed tumor growth and improved survival of the animals. Enhanced tumor infiltration by CD8+ T cells with increased effector function was observed, and depletion of CD8+ T cells abrogated the GITRL-mediated delay in tumor growth. Moreover, GITRL expression on tumor cells enhanced proliferation and reduced the numbers of apoptotic CD4+ and CD8+ T cells in vitro [50]. Piao and coworkers injected GITRL-transfected cell lines of different origin and strain and the respective parental controls into syngeneic mice. They found that the parental tumors grew progressively, while GITRL-expressing tumors regressed, and rechallenge of mice previously injected with GITRL-expressing cancer cells with the parental cell lines did not result in tumor growth. This study also confirmed the central role of CD8+ T cells for the growth reduction observed with GITRL-expressing tumor cells. Interestingly, the authors demonstrated that Treg contribute to tolerance to GITRL-negative tumors, while in a setting with GITRL-expressing tumor cells, CD8+ T cell effector functions overcome their regulatory function [42]. Nishikawa and coworkers immunized BALB/c mice with plasmids encoding a tumor rejection antigen and GITRL. They observed a 10-fold increase in the numbers of specific T cells and demonstrated that GITR signaling directly acted on CD8+ T cells, and, in a CMS5 sarcoma model, GITRL inhibited tumor growth which was dependent on the presence of CD8+, but not CD4+ T cells. Moreover, the investigators provided evidence that GITR signaling renders CD8+ T cells resistant to suppression by Treg [74]. Hu et al. utilized recombinant GITRL-Ig fusion protein, which they found to induce proliferation of CD8+ T cells and Treg, the latter thereupon loosing their suppressive phenotype. Moreover, application of GITRL-Ig caused regression of RENCA and Colon26 tumors in BALB/c mice. Depletion studies revealed the relevance of CD8+ T cells for the observed effects and an increased number of nonregulatory T cells among tumor-infiltrating lymphocytes were observed in GITRL-Ig treated animals [75]. Similar results were observed by these investigators upon treatment with agonistic GITR antibody, and meanwhile also multiple other studies have demonstrated that, beyond enforced GITRL expression or addition of recombinant GITRL, treatment with agonistic GITR antibody enhances antitumor immunity in mice. Ko et al. reported that intravenous or intratumoral administration of DTA-1 anti-GITR antibody eradicated established fibrosarcoma in BALB/c mice without eliciting substantial autoimmunity. Moreover, mice that had rejected tumors upon DTA-1 treatment rejected tumors upon rechallenge with even 10-fold higher tumor cell numbers indicating that the mice had developed specific antitumor immunity. In addition, tumors of mice treated with anti-GITR displayed large numbers of infiltrating activated T cells, and DTA-1 treatment increased the number of IFN-*γ* secreting T cells, which was required for tumor rejection [76]. DTA-1 treatment at the time of inoculation of B16 tumor cells into C57BL/6 had already been reported by Turk and coworkers to cause rejection upon a secondary challenge with the same tumor in the first published study suggesting that GITR could be a target for tumor therapy [77]. In another study, the same group employed combined vaccination with cancer self-antigens and anti-GITR antibody application for analysis of protection from B16 tumor challenge in C57BL/6 mice and addressed, among others, the effects of GITR triggering on CD8+ T cell responses [78]. Enhanced primary and recall CD8+ T cell responses like increased cell number, granule mobilization, and IFN-*γ* production were observed upon GITR activation during immunization with melanoma differentiation antigens, and these effects were only partially dependent on CD4+ T cells. GITR stimulation was associated with enhanced antitumor immunity, but also resulted in slight autoimmunity (i.e., hypopigmentation) in this study. Of note, the effects of anti-GITR application were found to be dependent on the time point of application during the immunization, which lends further evidence to the notion that the effects of GITR stimulation vary with the level of the immune response. Duan and coworkers found that treatment of C57BL/6 mice with agonistic GITR antibody led to regression of B16 tumors transfected to express a mutated self-antigen, and a marked tumor infiltration and enhanced numbers of CD8+ T cells specific for the mutated antigen were observed [79]. Other investigators studied the effects of anti-GITR on effector functions of CD8+ T cells from DUC18 donors adoptively transferred into BALB/c mice carrying CMS5 fibrosarcoma xenotransplants. They reported enhanced tumor infiltration, activation, proliferation, cytokine production, granule mobilization, and lytic activity [80]. Ramirez-Montagut and coworkers observed increased survival upon DTA-1 application in their tumor models (BALB/c mice injected with RENCA cells and C57BL/6 mice inoculated with B16 melanoma cells), which required presence of CD4+ and CD8+ T cells. These investigators further demonstrated that CD4+ T cells, CD8+ T cells, or a combination of both, derived from DTA-1 treated tumor-challenged mice, led to tumor rejection following adoptive transfer in their model system. They confirmed the important role of IFN-*γ* for DTA-1-mediated tumor rejection and provided evidence that CD178/Fas Ligand, but not perforin is required for GITR-mediated effects on antitumor immunity. In addition, this group also reported generation of T cell memory, mild signs of autoimmunity in a subset of treated animals, and enhanced activation and numbers of effector T cells, but also elevated numbers of Treg. It is of importance that in this study application of agonistic anti-GITR, besides modulating T cell reactivity, was also shown to influence the reactivity of other cell populations involved in antitumor immunity. Mice depleted of NK/NKT cells developed tumors rapidly, and application of DTA-1 had only little effect. This indicates that beyond T cells NK and/or NKT cell activity substantially contributed to the immune response induced by GITR stimulation [25]. An effect of agonistic anti-GITR on immune effector cells other than T cells was also observed by Zhou et al. They reported increased numbers and activation of T cells, but also of B cells and NK cells in draining lymph nodes of CT26 tumor-bearing BALB/c mice after DTA-1 treatment, and GITR stimulation enhanced PMA/Ionomycin-induced NK cell granule mobilization. DTA-1 also increased Treg numbers and activation, and no reduction of Treg suppressive capacity but resistance of responder CD4+ T cells to Treg suppression after in vivo GITR stimulation was observed. They further performed depletion experiments and reported that tumors grew faster in CD8+ T cell and NK cell depleted mice. DTA-1 treatment still mediated significant tumor rejection indicating that neither CD8+ nor NK cells were absolutely required for its efficacy. However, combined NK and CD8+ T cell depletion significantly compromised the effects of DTA-1 treatment, which led the investigators to conclude that there may be a redundant mechanism in tumor killing by these two cell types. In contrast, CD4+ T cell depletion completely abrogated the effects of DTA-1 on tumor growth, but also on activation of CD8+ T cells and NK cells. When interpreting these data, one should consider that the depletion strategy employed in this study may not have completely eradicated the respective cell types, as for example, only about 80% of NK cells, were successfully depleted [28]. Nevertheless, these data clearly indicate that various different immune effector cell types interact upon and contribute to effects of anti-GITR treatment in tumor-bearing mice. 

While most studies attributed the effects of DTA-1 treatment in tumor models to the agonistic properties of this antibody, two recent studies demonstrate that the impact of DTA-1 treatment in tumor models may also be due to effects other than GITR triggering. Coe et al. confirmed the potent antitumor effects of DTA-1 in C57BL/6 mice xenotransplanted with MB49 bladder carcinoma and found in ex vivo analyses that Treg of antibody-treated animals did not display reduced suppressive capacity as compared to Treg of untreated animals. Using foxp3/GFP knock-in mice they reported a reduction of tumor-infiltrating and circulating Treg in antibody treated animals. Moreover, they also observed depletion of monoclonal Treg adoptively transferred into Thy1.2B6 mice upon DTA-1 treatment with a more pronounced effect on tumor-infiltrating Treg compared to Treg in draining lymph nodes. GITR expression was more pronounced on Treg as compared to effector T cells and on Treg in tumors as compared to Treg of tumor draining lymph nodes, which led the authors to conclude that increased GITR expression of tumor-infiltrating Treg rendered them more susceptible to DTA-1 depletion. Thus, although DTA-1 was described as nondepleting by other investigators [15, 76], these results indicate that DTA-1 may be a depleting antibody in vivo which preferentially targets Treg due to their high levels of GITR expression, and that this mechanism contributes to its antitumor efficacy [81]. Cohen and coworkers demonstrated by analyses in C57BL/6 mice inoculated with B16 melanoma cells that DTA-1 treatment is more effective when given several days after tumor inoculation as compared to antibody treatment and tumor inoculation at the same day, suggesting that the efficacy of DTA-1 requires upregulation of GITR on tumor-activated T cells. They further reported that GITR triggering did not systemically alter the capacity of Treg or effector T cells to suppress or to be suppressed. When studying T cell subsets, they observed a decrease of the Treg frequency within the tumors following DTA-1 treatment resulting in an altered effector:regulatory T cell ratio, while this did not occur in spleens or tumor-draining lymph nodes. Analyses with adoptively transferred melanoma-specific T cells revealed that DTA-1 treatment reduced the accumulation of GITR-expressing, but not GITR-negative Treg in the tumor, while numbers in spleen and draining lymph nodes were not altered. Moreover, cells trafficking into the tumor displayed enhanced effector functions. Using foxp3/GFP mice they further found that DTA-1 treatment resulted in reduced Treg foxp3 expression in the tumor, which is indicative for loss of suppressive function. Finally, the authors provided evidence that lack of GITR expression on Treg or effector T cells attenuated the antitumor effect of DTA-1, while lack of GITR expression on both cell populations abrogated its effects [82]. Beyond confirming that GITR expression levels and depletion of GITR-bearing cells influence the antitumor effects of DTA-1, the data from these and other studies indicate that the optimal timing of GITR ligation may vary in different tumor models according to strain and immunogenicity and aggressiveness of the tumor. However, it remains yet unclear whether and how depletion of other GITR-bearing immune effector cells like B cells, NK cells and monocytes/macrophages upon DTA-1 treatment influences antitumor immunity. 

While the results obtained in the different studies vary substantially, the overall notion based on the data obtained in the murine system was that stimulation of GITR may be a promising approach for treatment of human cancers. However, data by us and others on the role of GITR in human NK cells indicate that stimulation of GITR may also impair antitumor immunity [11, 31, 33, 43]. Ex vivo analyses of human tumors from different histological origin and leukemic blasts revealed substantial expression of GITRL, which was not found in corresponding healthy tissues. In addition, sera from cancer patients but not from healthy donors contain substantial levels of sGITRL. Interaction of tumor-derived GITRL with NK-expressed GITR not only directly reduced NK cytotoxicity and IFN-*γ* production, but also the release of immunoinhibitory cytokines by tumor cells following reciprocal GITR-GITRL interaction impaired NK cell antitumor reactivity [11, 31, 43]. Recently we found evidence for another mechanism by which GITR-GITRL interaction may negatively affect antitumor immunity in humans. Available data indicate that platelets can increase metastasis by enabling tumor evasion from NK-mediated immune surveillance, but the understanding of the underlying molecular mechanisms was yet fragmentary at best [83, 84]. We observed that cancer cells of different origins (e.g., melanoma, prostate) rapidly get coated by GITRL-expressing platelets, which confers a seemingly GITRL-positive phenotype. This “GITRL pseudoexpression” on platelet-coated tumor cells substantially impairs NK cell reactivity ([44] and unpublished data). Thus it seems that expression/pseudoexpression of GITRL may enable tumor cells to evade immune surveillance by human NK cells, while a great body of data points to a stimulatory role of GITR triggering in antitumor immunity in mice. In this context it is important to bear in mind that species-specific differences following GITR triggering may not only occur with NK cells. One should also reconsider the available data with Treg, as suppression of human Treg, in contrast to their counterparts in mice, does not seem to be inhibited by GITR [47, 48]. Valuation of the role of GITR in antitumor immunity thus is complicated because GITR expression and function may depend on the time point and level of an ongoing immune response in general as well as on cellular activity and the respective cell type in particular. Moreover, while GITR has convincingly been shown to costimulate effector T cells, T cells from GITR^−/−^ mice have been shown to be hyperresponsive to immobilized anti-CD3 (e.g., references [14, 15, 45, 54]). In addition, both proapoptotic and antiapoptotic effects relying on modulation of, for example, Siva (in a Cos7 cell model) or the CD95/Fas pathway (in T cells) have been reported after GITR stimulation, which again seem to be dependent on the activation state and the biological environment (reviewed in [85]). 

The various signal transduction pathways modulated by GITR are yet not fully understood (for an excellent review see [86]). GITR signaling has been shown to involve five of the six mammalian TRAF proteins identified to date, and activation of the different TRAF molecules can result in varying and even opposite effects, which may, in addition, differ between mice and men [1, 2, 87–89]. For example, the two groups that initially identified human GITR reported that it positively influenced NF-*κ*B activation via the TRAF2/NIK pathway while downregulating NF-*κ*B activity via TRAF1 and TRAF3 [1, 2]. Esparza and coworkers reported, quite in contrast, that murine GITR coexpressed with TRAF2 reduced NF-*κ*B activation [88]. In this context, the recently reported difference in oligomerization between human and murine GITRL may also be relevant. It may result in recruitment of different adaptor molecules, which in turn could explain species-specific effects of GITR-GITRL interaction [7–10]. The interpretation of available data on the function of the GITR-GITRL molecule system and its modulation in antitumor immunity becomes even more difficult due to the fact that both GITR and GITRL are expressed by multiple cell types and can both transduce signals (Figure 1). Therapeutic intervention thus affects the reactivity of various different immune effector cells involved in the antitumor immune response, and the consequences of GITR-GITRL interaction may vary depending on the involved cell types. Moreover, the effects of therapeutically modulating GITR by antibodies like DTA-1 or recombinant ligand do not reflect the consequences of GITR interaction with its natural, tumor-expressed ligand in vivo. This may explain some of the seemingly contradicting results observed in different studies.

## 7. Conclusion

Initially considered an inhibitor of Treg activity, GITR has meanwhile been shown to affect multiple cell types. Depletion experiments can only partially serve to explain the mechanisms and consequences of therapeutic GITR stimulation, as the lack of a certain effector cell population may influence the complex crosstalk of the different components in antitumor immunity. This is even more since bidirectional signals are mediated following GITR-GITRL interaction, and studies in GITR^−/−^ mice have not yet led to a clear picture of the role of this molecule system in normal physiology. In addition, the available results regarding the consequences of GITR stimulation on different T cell subsets and with different tumor models have been found to be influenced by the time of intervention, the biological environment, and the level of the ongoing immune response. Moreover, they may depend on the aggressiveness of the respective tumor models. Maybe most important with regard to the translation of findings obtained in mouse models into clinical treatment strategies, effects of GITR may differ between mice and men. Additional studies are required to define the conditions upon which therapeutic GITR stimulation activates antitumor immunity in humans, before further steps to use GITR/GITRL-modulating reagents for therapy of cancer patients should be undertaken.

## References

[1] Gurney AL, Marsters SA, Huang A (1999). Identification of a new member of the tumor necrosis factor family and its receptor, a human ortholog of mouse GITR. *Current Biology*.

[2] Kwon B, Yu K-Y, Ni J (1999). Identification of a novel activation-inducible protein of the tumor necrosis factor receptor superfamily and its ligand. *The Journal of Biological Chemistry*.

[3] Nocentini G, Giunchi L, Ronchetti S (1997). A new member of the tumor necrosis factor/nerve growth factor receptor family inhibits T cell receptor-induced apoptosis. *Proceedings of the National Academy of Sciences of the United States of America*.

[4] Nocentini G, Ronchetti S, Bartoli A (2000). Identification of three novel mRNA splice variants of GITR. *Cell Death and Differentiation*.

[5] Kim JD, Choi BK, Bae JS (2003). Cloning and characterization of GITR ligand. *Genes and Immunity*.

[6] Tone M, Tone Y, Adams E (2003). Mouse glucocorticoid-induced tumor necrosis factor receptor ligand is costimulatory for T cells. *Proceedings of the National Academy of Sciences of the United States of America*.

[7] Chattopadhyay K, Ramagopal UA, Mukhopadhaya A (2007). Assembly and structural properties of glucocorticoid-induced TNF receptor ligand: implications for function. *Proceedings of the National Academy of Sciences of the United States of America*.

[8] Chattopadhyay K, Ramagopal UA, Brenowitz M, Nathenson SG, Almo SC (2008). Evolution of GITRL immune function: murine GITRL exhibits unique structural and biochemical properties within the TNF superfamily. *Proceedings of the National Academy of Sciences of the United States of America*.

[9] Zhou Z, Song X, Berezov A (2008). Human glucocorticoid-induced TNF receptor ligand regulates its signaling activity through multiple oligomerization states. *Proceedings of the National Academy of Sciences of the United States of America*.

[10] Zhou Z, Tone Y, Song X (2008). Structural basis for ligand-mediated mouse GITR activation. *Proceedings of the National Academy of Sciences of the United States of America*.

[11] Baltz KM, Krusch M, Baessler T (2008). Neutralization of tumor-derived soluble Glucocorticoid-Induced TNFR-related protein ligand increases NK cell anti-tumor reactivity. *Blood*.

[12] Baumgartner-Nielsen J, Vestergaard C, Thestrup-Pedersen K, Deleuran M, Deleuran B (2006). Glucocorticoid-induced tumour necrosis factor receptor (GITR) and its ligand (GITRL) in atopic dermatitis. *Acta Dermato-Venereologica*.

[13] Mahesh SP, Li Z, Liu B, Fariss RN, Nussenblat RB (2006). Expression of GITR ligand abrogates immunosuppressive function of ocular tissue and differentially modulates inflammatory cytokines and chemokines. *European Journal of Immunology*.

[14] McHugh RS, Whitters MJ, Piccirillo CA (2002). CD4+CD25+ Immunoregulatory T Cells: gene expression analysis reveals a functional role for the glucocorticoid-induced TNF receptor. *Immunity*.

[15] Shimizu J, Yamazaki S, Takahashi T, Ishida Y, Sakaguchi S (2002). Stimulation of CD25+CD4+ regulatory T cells through GITR breaks immunological self-tolerance. *Nature Immunology*.

[16] Stephens GL, McHugh RS, Whitters MJ (2004). Engagement of glucocorticoid-induced TNFR family-related receptor on effector T cells by its ligand mediates resistance to suppression by CD4+CD25+ T cells. *Journal of Immunology*.

[17] Ikeda M, Takeshima F, Ohba K (2006). Flow cytometric analysis of expression of transforming growth factor-*β* and glucocorticoid-induced tumor necrosis factor receptor on CD4+CD25+ T cells of patients with inflammatory bowel disease. *Digestive Diseases and Sciences*.

[18] Li Z, Mahesh SP, Kim BJ, Buggage RR, Nussenblatt RB (2003). Expression of Glucocorticoid Induced TNF Receptor Family Related Protein (GITR) on peripheral T cells from normal human donors and patients with non-infectious uveitis. *Journal of Autoimmunity*.

[19] Kanamaru F, Youngnak P, Hashiguchi M (2004). Costimulation via glucocorticoid-induced TNF receptor in both conventional and CD25+ regulatory CD4+ T cells. *Journal of Immunology*.

[20] Kim J, Choi WS, Kang H (2006). Conversion of alloantigen-specific CD8+ T cell anergy to CD8+ T cell priming through in vivo ligation of glucocorticoid- induced TNF receptor. *Journal of Immunology*.

[21] Nishioka T, Nishida E, Iida R, Morita A, Shimizu J (2008). In vivo expansion of CD4+Foxp3+ regulatory T cells mediated by GITR molecules. *Immunology Letters*.

[22] Ronchetti S, Nocentini G, Bianchini R, Krausz LT, Migliorati G, Riccardi C (2007). Glucocorticoid-induced TNFR-related protein lowers the threshold of CD28 costimulation in CD8+ T cells. *Journal of Immunology*.

[23] Suvas S, Kim B, Sarangi PP, Tone M, Waldmann H, Rouse BT (2005). In vivo kinetics of GITR and GITR ligand expression and their functional significance in regulating viral immunopathology. *Journal of Virology*.

[24] Kohm AP, Williams JS, Miller SD (2004). Cutting edge: ligation of the glucocorticoid-Induced TNF receptor enhances autoreactive CD4+ T cell activation and experimental autoimmune encephalomyelitis. *Journal of Immunology*.

[25] Ramirez-Montagut T, Chow A, Hirschhorn-Cymerman D (2006). Glucocorticoid-induced TNF receptor family related gene activation overcomes tolerance/ignorance to melanoma differentiation antigens and enhances antitumor immunity. *Journal of Immunology*.

[26] Chen S, Ndhlovu LC, Takahashi T (2008). Co-inhibitory roles for glucocorticoid-induced TNF receptor in CD1d-dependent natural killer T cells. *European Journal of Immunology*.

[27] Kim W-J, Bae E-M, Kang Y-J (2006). Glucocorticoid-induced tumour necrosis factor receptor family related protein (GITR) mediates inflammatory activation of macrophages that can destabilize atherosclerotic plaques. *Immunology*.

[28] Zhou P, L’Italien L, Hodges D, Schebye XM (2007). Pivotal roles of CD4+ effector T cells in mediating agonistic anti-GITR mAb-induced-immune activation and tumor immunity in CT26 tumors. *Journal of Immunology*.

[29] Bae E, Kim W-J, Kang Y-M (2007). Glucocorticoid-induced tumour necrosis factor receptor-related protein-mediated macrophage stimulation may induce cellular adhesion and cytokine expression in rheumatoid arthritis. *Clinical and Experimental Immunology*.

[30] Bae EM, Kim W-J, Suk K (2008). Reverse signaling initiated from GITRL induces NF-*κ*B activation through ERK in the inflammatory activation of macrophages. *Molecular Immunology*.

[31] Baltz KM, Krusch M, Bringmann A (2007). Cancer immunoediting by GITR (glucocorticoid-induced TNF-related protein) ligand in humans: NK cell/tumor cell interactions. *The FASEB Journal*.

[32] Hanabuchi S, Watanabe N, Wang Y-H (2006). Human plasmacytoid predendritic cells activate NK cells through glucocorticoid-induced tumor necrosis factor receptor-ligand (GITRL). *Blood*.

[33] Liu B, Li Z, Mahesh SP (2008). Glucocorticoid-induced tumor necrosis factor receptor negatively regulates activation of human primary natural killer (NK) cells by blocking proliferative signals and increasing NK cell apoptosis. *The Journal of Biological Chemistry*.

[34] Wang J, Devgan V, Corrado M (2005). Glucocorticoid-induced tumor necrosis factor receptor is a p21 Cip1/WAF1 transcriptional target conferring resistance of keratinocytes to UV light-induced apoptosis. *The Journal of Biological Chemistry*.

[35] Lahey TP, Loisel SD, Wieland-Alter W (2007). Glucocorticoid-induced tumor necrosis factor receptor family-related protein triggering enhances HIV-specific CD4+ T cell cytokine secretion and protects HIV-specific CD4+ T cells from apoptosis. *Journal of Infectious Diseases*.

[36] Wilde B, Dolff S, Cai X (2009). CD4+CD25+ T-cell populations expressing CD134 and GITR are associated with disease activity in patients with Wegener’s granulomatosis. *Nephrology Dialysis Transplantation*.

[37] Lee J-H, Wang L-C, Lin Y-T, Yang Y-H, Lin D-T, Chiang B-L (2006). Inverse correlation between CD4+ regulatory T-cell population and autoantibody levels in paediatric patients with systemic lupus erythematosus. *Immunology*.

[38] Łuczyński W, Wawrusiewicz-Kurylonek N, Stasiak-Barmuta A (2009). Diminished expression of ICOS, GITR and CTLA-4 at the mRNA level in T regulatory cells of children with newly diagnosed type 1 diabetes. *Acta Biochimica Polonica*.

[39] Kim BJ, Li Z, Fariss RN (2004). Constitutive and cytokine-induced GITR ligand expression on human retinal pigment epithelium and photoreceptors. *Investigative Ophthalmology and Visual Science*.

[40] Cardona ID, Goleva E, Ou L-S, Leung DYM (2006). Staphylococcal enterotoxin B inhibits regulatory T cells by inducing glucocorticoid-induced TNF receptor-related protein ligand on monocytes. *Journal of Allergy and Clinical Immunology*.

[41] Hwang H, Lee S, Lee W-H, Lee H-J, Suk K (2010). Stimulation of glucocorticoid-induced tumor necrosis factor receptor family-related protein ligand (GITRL) induces inflammatory activation of microglia in culture. *Journal of Neuroscience Research*.

[42] Piao J, Kamimura Y, Iwai H (2009). Enhancement of T-cell-mediated anti-tumour immunity via the ectopically expressed glucocorticoid-induced tumour necrosis factor receptor-related receptor ligand (GITRL) on tumours. *Immunology*.

[43] Baessler T, Krusch M, Schmiedel BJ (2009). Glucocorticoid-induced tumor necrosis factor receptor-related protein ligand subverts immunosurveillance of acute myeloid leukemia in humans. *Cancer Research*.

[44] Placke T, Kopp HG, Kanz L (2009). Coating of tumor cells by platelets confers expression of immunoregulatory molecules which impair NK cell anti-tumor reactivity. *Blood*.

[45] Ji H-B, Liao G, Faubion WA (2004). Cutting edge: the natural ligand for glucocorticoid-induced TNF receptor-related protein abrogates regulatory T cell suppression. *Journal of Immunology*.

[46] Ronchetti S, Zollo O, Bruscoli S (2004). GITR, a member of the TNF receptor superfamily, is costimulatory to mouse T lymphocyte subpopulations. *European Journal of Immunology*.

[47] Levings MK, Sangregorio R, Sartirana C (2002). Human CD25+CD4+ T suppressor cell clones produce transforming growth factor *β*, but not interleukin 10, and are distinct from type 1 T regulatory cells. *Journal of Experimental Medicine*.

[48] Tuyaerts S, Van Meirvenne S, Bonehill A (2007). Expression of human GITRL on myeloid dendritic cells enhances their immunostimulatory function but does not abrogate the suppressive effect of CD4+CD25+ regulatory T cells. *Journal of Leukocyte Biology*.

[49] Calmels B, Paul S, Futin N, Ledoux C, Stoeckel F, Acres B (2005). Bypassing tumor-associated immune suppression with recombinant adenovirus constructs expressing membrane bound or secreted GITR-L. *Cancer Gene Therapy*.

[50] Cho JS, Hsu JV, Morrison SL (2009). Localized expression of GITR-L in the tumor microenvironment promotes CD8+ T cell dependent anti-tumor immunity. *Cancer Immunology, Immunotherapy*.

[51] Esparza EM, Arch RH (2005). Glucocorticoid-induced TNF receptor functions as a costimulatory receptor that promotes survival in early phases of T cell activation. *Journal of Immunology*.

[52] Igarashi H, Cao Y, Iwai H (2008). GITR ligand-costimulation activates effector and regulatory functions of CD4+ T cells. *Biochemical and Biophysical Research Communications*.

[53] Agostini M, Cenci E, Pericolini E (2005). The glucocorticoid-induced tumor necrosis factor receptor-related gene modulates the response to Candida albicans infection. *Infection and Immunity*.

[54] Ronchetti S, Nocentini G, Riccardi C, Pandolfi PP (2002). Role of GITR in activation response of T lymphocytes. *Blood*.

[55] Cuzzocrea S, Ayroldi E, Di Paola R (2005). Role of glucocorticoid-induced TNF receptor family gene (GITR) in collagen-induced arthritis. *The FASEB Journal*.

[56] van Olffen RW, Koning N, van Gisbergen KP (2009). GITR triggering induces expansion of both effector and regulatory CD4+ T cells in vivo. *Journal of immunology*.

[57] Cuzzocrea S, Nocentini G, Di Paola R (2006). Proinflammatory role of glucocorticoid-induced TNF receptor-related gene in acute lung inflammation. *Journal of Immunology*.

[58] Cuzzocrea S, Ronchetti S, Genovese T (2007). Genetic and pharmacological inhibition of GITR-GITRL interaction reduces chronic lung injury induced by bleomycin instillation. *The FASEB Journal*.

[59] Patel M, Xu D, Kewin P (2005). Glucocorticoid-induced TNFR family-related protein (GITR) activation exacerbates murine asthma and collagen-induced arthritis. *European Journal of Immunology*.

[60] You S, Poulton L, Cobbold S (2009). Key role of the GITR/GITRLigand pathway in the development of murine autoimmune diabetes: a potential therapeutic target. *PLoS ONE*.

[61] La S, Kim E, Kwon B (2005). In vivo ligation of glucocorticoid-induced TNF receptor enhances the T-cell immunity to herpes simplex virus type 1. *Experimental and Molecular Medicine*.

[62] Haque A, Stanley AC, Amante FH (2010). Therapeutic glucocorticoid-induced TNF receptor-mediated amplification of CD4+ T cell responses enhances antiparasitic immunity. *Journal of Immunology*.

[63] Bushell A, Wood K (2007). GITR ligation blocks allograft protection by induced CD25+CD4+ regulatory T cells without enhancing effector T-cell function. *American Journal of Transplantation*.

[64] Kim J, Woon SC, Hye JK, Kwon B (2006). Prevention of chronic graft-versus-host disease by stimulation with glucocorticoid-induced TNF receptor. *Experimental and Molecular Medicine*.

[65] Muriglan SJ, Ramirez-Montagut T, Alpdogan O (2004). GITR activation induces an opposite effect on alloreactive CD4+ and CD8+ T cells in graft-versus-host disease. *Journal of Experimental Medicine*.

[66] Shevach EM, Stephens GL (2006). Opinion: the GITR-GITRL interaction: co-stimulation or contrasuppression of regulatory activity?. *Nature Reviews Immunology*.

[67] Smith CA, Farrah T, Goodwin RG (1994). The TNF receptor superfamily of cellular and viral proteins: activation, costimulation, and death. *Cell*.

[68] Grohmann U, Volpi C, Fallarino F (2007). Reverse signaling through GITR ligand enables dexamethasone to activate IDO in allergy. *Nature Medicine*.

[69] Vecchiarelli A, Pericolini E, Gabrielli E (2009). The GITRL-GITR system alters TLR-4 expression on DC during fungal infection. *Cellular Immunology*.

[70] Lee H-S, Shin H-H, Kwon BS, Choi H-S (2003). Soluble glucocorticoid-induced tumor necrosis factor receptor (sGITR) increased MMP-9 activity in murine macrophage. *Journal of Cellular Biochemistry*.

[71] Shin H-H, Lee M-H, Kim S-G, Lee Y-H, Kwon BS, Choi H-S (2002). Recombinant glucocorticoid induced tumor necrosis factor receptor (rGITR) induces NOS in murine macrophage. *FEBS Letters*.

[72] Shin H-H, Kwon BS, Choi H-S (2002). Recombinant glucocorticoid induced tumour necrosis factor receptor (rGITR) induced COX-2 activity in murine macrophage Raw 264.7 cells. *Cytokine*.

[73] Shin H-H, Kim S-J, Lee H-S, Choi H-S (2004). The soluble glucocorticoid-induced tumor necrosis factor receptor causes cell cycle arrest and apoptosis in murine macrophages. *Biochemical and Biophysical Research Communications*.

[74] Nishikawa H, Kato T, Hirayama M (2008). Regulatory T cell-resistant CD4+ T cells induced by glucocorticoid-induced tumor necrosis factor receptor signaling. *Cancer Research*.

[75] Hu P, Arias RS, Sadun RE (2008). Construction and preclinical characterization of Fc-mGITRL for the immunotherapy of cancer. *Clinical Cancer Research*.

[76] Ko K, Yamazaki S, Nakamura K (2005). Treatment of advanced tumors with agonistic anti-GITR mAb and its effects on tumor-infiltrating Foxp3+CD25+CD4+ regulatory T cells. *Journal of Experimental Medicine*.

[77] Turk MJ, Guevara-Patiño JA, Rizzuto GA, Engelhorn ME, Houghton AN (2004). Concomitant tumor immunity to a poorly immunogenic melanoma is prevented by regulatory T cells. *Journal of Experimental Medicine*.

[78] Cohen AD, Diab A, Perales M-A (2006). Agonist anti-GITR antibody enhances vaccine-induced CD8+ T-cell responses and tumor immunity. *Cancer Research*.

[79] Duan F, Lin Y, Liu C (2009). Immune rejection of mouse tumors expressing Mutated self. *Cancer Research*.

[80] Imai N, Ikeda H, Tawara I (2009). Glucocorticoid-induced tumor necrosis factor receptor stimulation enhances the multifunctionality of adoptively transferred tumor antigen-specific CD8+ T cells with tumor regression. *Cancer Science*.

[81] Coe D, Begom S, Addey C, White M, Dyson J, Chai J-G (2010). Depletion of regulatory T cells by anti-GITR mAb as a novel mechanism for cancer immunotherapy. *Cancer Immunology, Immunotherapy*.

[82] A. D. Cohen, Schaer DA, Liu C (2010). Agonist anti-GITR monoclonal antibody induces melanoma tumor immunity in mice by altering regulatory T cell stability and intra-tumor accumulation. *PLoS One*.

[83] Jin DK, Shido K, Kopp H-G (2006). Cytokine-mediated deployment of SDF-1 induces revascularization through recruitment of CXCR4+ hemangiocytes. *Nature Medicine*.

[84] Palumbo JS, Talmage KE, Massari JV (2005). Platelets and fibrin(ogen) increase metastatic potential by impeding natural killer cell-mediated elimination of tumor cells. *Blood*.

[85] Nocentini G, Riccardi C (2005). GITR: a multifaceted regulator of immunity belonging to the tumor necrosis factor receptor superfamily. *European Journal of Immunology*.

[86] Krausz LT, Bianchini R, Ronchetti S, Fettucciari K, Nocentini G, Riccardi C (2007). GITR-GITRL system, a novel player in shock and inflammation. *TheScientificWorldJournal*.

[87] Esparza EM, Arch RH (2004). TRAF4 functions as an intermediate of GITR-induced NF-*κ*B activation. *Cellular and Molecular Life Sciences*.

[88] Esparza EM, Arch RH (2005). Glucocorticoid-induced TNF receptor, a costimulatory receptor on naive and activated T cells, uses TNF receptor-associated factor 2 in a novel fashion as an inhibitor of NF-*κ*B activation. *Journal of Immunology*.

[89] Esparza EM, Lindsten T, Stockhausen JM, Arch RH (2006). Tumor necrosis factor receptor (TNFR)-associated factor 5 is a critical intermediate of costimulatory signaling pathways triggered by glucocorticoid-induced TNFR in T cells. *The Journal of Biological Chemistry*.

